# Impact of Innovative Emotion Training in Preschool and Kindergarten Children Aged from 3 to 6 Years

**DOI:** 10.3390/children10111825

**Published:** 2023-11-17

**Authors:** Anne Lafay, Carole Berger, Laura Alaria, Sonia Angonin, Nathalie Dalla-Libera, Sylvie Richard, Thalia Cavadini, Edouard Gentaz

**Affiliations:** 1 Department of Psychology, Univ. Grenoble Alpes, Univ. Savoie Mont Blanc, Centre Nationale de la Recherche Scientifique (CNRS), Laboratoire de Psychologie et NeuroCognition (LPNC), 38000 Grenoble, France; carole.berger@univ-smb.fr (C.B.); laura.alaria@univ-smb.fr (L.A.); 2 French Ministry of National Education, 75007 Paris, France; sonia.angonin@ac-grenoble.fr (S.A.); nathalie.dalla-libera@ac-grenoble.fr (N.D.-L.); 3 Faculty of Psychology and Educational Science, University of Geneva, 1205 Geneva, Switzerland; sylvie.richard@hepvs.ch (S.R.); thalia.cavadini@unige.ch (T.C.); edouard.gentaz@unige.ch (E.G.); 4 Department of Psychology, Valais University of Teacher Education, Haute Ecole Pédagogique du Valais (HEP-VS), 1890 Saint-Maurice, Switzerland

**Keywords:** emotion, children, preschool, kindergarten, training, transfer, language, mathematics

## Abstract

Children’s emotional abilities have been shown to be related to academic performance, peer acceptance, and in-school adjustment. The main objective of this study was to evaluate the effect of innovative emotion training designed to promote the emotional abilities of 316 preschool/kindergarten children aged from 3 to 6 years old enrolled in public schools in the first three levels (L1, L2, and L3). Another objective was to examine the transfer effects on language comprehension and mathematics abilities. The emotion training (eight sessions) focused on the identification, comprehension, and expression of emotions and were co-constructed with teachers. Children were tested before and after the training on emotion, language, and mathematics skills. Results showed an improvement in emotional abilities in young children of L1 (3–4 years) and L2 (4–5 years) in the intervention group compared to those in the non-intervention group. Also, although children’s emotion basic abilities were correlated with their language comprehension and mathematics abilities, the nature of this link was not demonstrated to be causal. Findings are discussed in regard to the influence of the level and in regard to links with academic variables.

## 1. Introduction

A recent large-scale meta-analysis showed a significant link between student’s emotional abilities and school performance from elementary to university levels [1]. The ability models of emotional intelligence (EI) conceptualize EI as a cognitive ability. The following four main abilities were generally identified [2]: (a) emotional perception, (b) ability to integrate emotional information and then use it as an input or support in cognitive tasks or decisions, (c) comprehension of emotions, and (d) emotional regulation for a specific goal. 

Three underlining mechanisms were proposed to explain this correlational link between emotional abilities and academic performance [1]: (1) the first mechanism is based on the ability to build social relationships at school (emotional abilities contribute to managing social world and establishing relationships with teachers, peers, and family); (2) a second mechanism is based on the ability to regulate emotions at school (and especially on the ability to regulate negative emotions); and (3) a third mechanism corresponds to the overlap that exists between academic content and emotional abilities. However, this large-scale meta-analysis did not consider the studies in preschool and kindergarten children.

Several studies have focused on the abilities that prepare children for school, and these are the foundation for learning and progress. Indeed, it is during the first years of school that children develop skills that contribute to their future academic success [3]. Among these abilities, “emotion knowledge” significantly contributes. Emotional knowledge in young children corresponds to the ability to recognize emotions, label facial expressions (that convey emotions), and identify behavioral cues and social contexts that generate emotions [4,5,6]. Voltmer and von Salisch [7] conducted a meta-analysis of studies involving children aged 3 to 12 years. They reported that a higher level of emotional ability tended to be associated with better academic achievement, peer acceptance, and school adjustment. At the first level, emotional abilities significantly predicted academic achievement. This was assessed through different tasks: “The Letter-Word” identification task, “The Dictation” task, and a task that measured practical mathematics problem-solving skills [8]. Positive correlations have also been reported between emotional abilities and school achievement in preschoolers when concept knowledge and language (the use of nouns and verbs) are considered [9].

Recently, Cavadini et al. [10] showed that emotional abilities, locomotor activity, social behavior, and mathematics performance are interrelated in 706 French preschool/kindergarten children aged 3 to 6 (children were tested as early as possible in their educational curriculum from levels 1 to 3). Because emotional abilities involve many components, each child’s total score was obtained in this study by summing up the scores of the following three tasks: (a) recognizing the primary emotions of anger, fear, joy, and sadness as well as a neutral facial expression (task 1: identification), (b) understanding the external causes of these emotions by pointing out which emotion (among five) a character felt in several given situations (task 2: understanding), (c) understanding external causes by naming each emotion correctly (task 3: denomination). Analyses revealed that higher mathematics performance was explained by higher emotional abilities and social behavior. In turn, children with a greater comprehension of emotions also tended to have higher scores in mathematics and locomotion tasks.

Regarding the emotional ability assessed in Cavadini et al.’s study [10], it has already been shown that this ability is involved in the regulation of emotions and behavior [11], which impacts children’s academic performance [12], and which also impacts the development of relevant and adaptive social interactions during early schooling [13]. Indeed, relations between emotional and social abilities have been highlighted in several studies or meta-analyses [6,14]. In line with these studies, the results of Cavadini et al. [10] supported the idea that social behavior has a mediation role between emotional abilities and mathematics performance.

MacCann et al. [1] concluded their meta-analysis by highlighting the main limitation of these cross-sectional studies, which do not provide information on the causal direction (pp. 173–174): “*The results revealed only that EI and academic performance are significantly associated, but not that higher EI causes higher achievement. This association could occur because (a) higher EI causes increased academic performance, (b) higher achievement causes increased EI, or (c) there are one or more variables that influence both EI and academic performance. (…) It seems likely that the reality is complex, with bidirectional effects of academic and emotional development, particularly in the earlier years of school*”. 

To further investigate the causal relationship between emotional abilities and academic achievement, and because the ultimate objective is to improve teaching practices in classrooms, training on emotional abilities was co-constructed by researchers and teachers. The classroom context appears to be relevant for training emotional abilities since school is a meeting place where children experience social relations and their complexity; schooling may then play a major role in the development of emotional abilities [15,16,17].

Daily pedagogical practices in the classroom bring many academic and social benefits [18]. Two direct practices of interest have been described as contributing to the development of these emotional abilities [19]. Firstly, to support children’s ability to identify emotions, the teacher must ensure that she/he regularly presents accurate words to name the emotions that are described either from external supports (images, videos) or from children’s own feelings. She/he must encourage and make explicit the gathering of clues from facial expressions, posture, verbalizations, thoughts, prosody, and corporeal and physiological clues (heart rate, physical sensations; [19]). These activities are related to the perception and understanding of emotions and can be conducted as early as 3 years old [20,21].

Secondly, in-class rich verbalization about emotions appears to be essential [11,22]. Talking about emotions could enhance children’s emotion comprehension, pro-social behaviors (helping, sharing), and inhibitory control in an emotional context [22,23,24]. Verbalization allows for the implicit knowledge of mental states to be made explicit through collaborative dialogue with peers or with an adult who supports this process [25,26,27]. According to Yelinek and Grady [28], in-class verbalization should include specific emotions (such as fear, anger, sadness, and joy), a description of the causes of emotions, and questions about these emotions. In fact, training conceptualized by the research team aimed to promote emotion and social abilities, demonstrating beneficial effects through peer dialogues conducted in 4- and 5-year-old children with special needs [29]. In particular, training used stories or vignettes that presented emotional issues in a way that children could understand; then, these stories were used to encourage children to discuss different subjects.

## 2. Current Study: Objective and Hypotheses

The current study aimed to assess whether co-constructed training with teachers on emotional abilities could improve children’s emotional abilities and whether it would also improve their mathematic and language abilities. The implementation of school training aimed at developing emotional abilities in typically developing young children involves both the consideration of scientific knowledge and its transfer in an intervention that is closely linked to the needs and practices of teachers and children. A collaborative approach then may help the transfer by promoting bridges between evidence-based data and professional practices in the French context [30].

Specifically, the main objective was to explore whether the training of emotional abilities in school (preschool and kindergarten) can impact children’s emotional abilities. We hypothesized that young children who benefited from emotional training could show a better development of their emotional abilities than children who had business-as-usual training (hypothesis 1). Exploratory analysis allowed us to deeply understand what emotion abilities and what emotion types improved after the training sessions.

Another objective was to investigate the link between children’s emotional abilities on the one hand and their language comprehension and mathematics abilities on the other hand. More specifically, we assessed whether children’s emotional abilities were correlated to their language comprehension and mathematics abilities. We assumed that we would replicate former results [10,31] and that children’s emotional abilities would be correlated with children’s mathematics abilities. We also expected that we would extend this result so that children’s emotional abilities would be correlated with language comprehension abilities (hypothesis 2). Previous studies already show an association between emotion and language comprehension in 3-years-olds [32] and between emotion and language expression in 3–5-year-old children [33]. Our study, therefore, brings more information about the link between emotion and language comprehension in 3–6-year-old children.

The third objective was to explore whether the training of emotional abilities in preschool/kindergarten could impact children’s language comprehension and mathematics abilities (the proactive transfer effect). The concept of transfer refers to the ability to apply the knowledge or abilities learned in a given situation to a new context: in other words, the ability to generalize what has been learned to a new situation [34,35]. The notion of proactive transfer indicates that elements of learning are re-used in subsequent learning (while that of retroactive transfer implies that learning influences previous learning). By improving this ability through training, one can examine whether it constitutes a basis for early learning. Our approach was then relevant to draw causal inferences, which the correctional technique does not allow. By referring to studies using mediation models [7,10], we proposed the hypothesis that emotional abilities are a determinant of early learning. If the link between emotional abilities, on the one hand, and language and mathematics abilities, on the other hand, is causal, we expected that young children who followed the emotional training would improve their language and mathematics abilities better than their peers who did not (hypothesis 3). Beyond the issue of these causal links between emotional abilities and early learning, the focus of this study was on whether relevant school training could improve children’s emotional abilities.

## 3. Materials and Methods

### 3.1. Participants

The schools were in a very large area of the Savoie department (France, 73). In total, 585 children aged from 3–6 years old participated as subjects as follows: 316 in the intervention group and 286 non-intervention group. Table 1 shows the demographic information of participants. They were enrolled in preschool/kindergarten public schools in the first level of the curriculum (Level 1: 3–4 years old), in the second level of the curriculum (Level 2: 4–5 years old), and in the third level of the curriculum (Level 3: 5–6 years old). The difference in gender distribution between the two groups (information missing for 21 children) was not significant for Level 1, χ^2^(1, *N* = 169) = 0.982, *p* = 0.554, for Level 2, χ^2^(1, *N* = 154) = 0.479, *p* = 0.293, or Level 3, χ^2^(1, *N* = 241) = 0.787, *p* = 0.445. For the difference in “Indice de Position Sociale” (IPS; [36]) related to the socioeconomic status, the interaction of Level × Group was significant, *F*(2579) = 8.949, *p* < 0.001, η^2^_p_ = 0.030. However, there was no significant difference in IPS between the intervention and the non-intervention groups at Levels 1 and 2; the intervention group had a higher IPS than the non-intervention group at Level 3. The IPS was integrated into the statistical analysis as a covariable.

### 3.2. Design and General Procedures

The project was approved by the Ethics Committee of the Université Savoie Mont Blanc. The study was a pretest—training—posttest design. The project was presented to the teachers, and they could participate on a voluntary basis. Children were assigned to one of the two groups. Because the training was administered by the teachers, the children of a whole classroom were all assigned to the same group for the function of teaching volunteering. First, an intervention group received training on emotional abilities from the volunteer teachers involved in the project. The emotional training was developed through collaborative work between researchers and teachers. Second, in a non-intervention group (business as usual), children and their teacher did not receive emotional training, and the teachers were said to conduct their classroom activities as they usually did (the school program included that children develop, at the end of kindergarten, the ability to identify and verbally express their emotions and feeling; this method is not indicated).

The assessment of emotion, mathematics, and language abilities was conducted in December (pretest, three months after the beginning of the school year) and five months later in May (posttest, after the training). The assessment took place at school and lasted 20–30 min. The experimenters started with the emotional abilities test and then administered mathematics and language tests.

Teachers and students (from the Department of Psychology at the Université Savoie Mont Blanc), who had been previously trained and continuously monitored by the researchers, assessed the children in schools. The assessments were administered in a typical classroom setting (with a preliminary pilot experiment to test our protocol in order to verify its feasibility and validity). A teacher assistant managed the classroom while the teacher or university student tested each pupil individually. Working together with the teachers in this way had two advantages. First, in order to test children’s emotional abilities, we were able to give them an experimental protocol that included the specific material, instructions, and scoring rules required for its implementation. Second, we could rely on official teaching aids by integrating some of their content into the current study in order to assess academic competencies and meet the preschool/kindergarten objectives (thus, we avoided disrupting the teaching curriculum for the classes involved in the study).

### 3.3. Innovative Emotion Training

The present research was conducted in close collaboration between the research team and a group of teachers. The aim was to develop training that was relevant given theoretical knowledge, and that could be fully integrated into the daily school life of children and teachers. During the preparation phase of this project (2020–2021), the teachers took part in several interactive workshops. The aims of these workshops were as follows: (a) to provide theoretical information on children’s emotional abilities (with focus on the development of these abilities and on their role in school learning), (b) to co-elaborate the content of each training session and the testing (material, activities, instructions), (c) to co-elaborate the structuration of the sequences, and finally (d) to train teachers and university students on the test administration in standardized ways. The tests were also co-constructed because children needed to be tested on what they were expected to progress in regarding the preschool/kindergarten curriculum. The workshops also offered the opportunity to share teaching experiences (especially when teachers started implementing training in their classrooms). At this point, before testing in 2021–2022 and in order to promote good implementation conditions, modifications were introduced, which were based on feedback from teachers (e.g., adequacy between activities and targeted abilities, children’s receptivity). 

The training phase consisted of three distinct sequences, which, respectively, focused on the following: (a) the identification of primary emotions (sequence 1), (b) the comprehension of the causes of one’s own emotions and those of others (sequence 2), and (c) the expression of emotions (sequence 3). The aim was to develop children’s emotional abilities using activities integrated into the classroom. Each sequence included different sessions, each of them with specific and structured activities developed in collaboration between teachers and researchers. All materials and the structure of the sessions were decided on in a collaborative way during workshops.

Sequence 1 (identification of emotions) included three sessions lasting 20–30 min each, which addressed the following, respectively: (a) the labeling of primary emotions, (b) the visual identification of facial expressions (from photos of multiethnic expressive faces), (c) the visual identification of body postures (from photos of characters in action presented with blurred faces in order to prevent the processing of facial signals). 

Sequence 2 (comprehension of the causes of emotions) included four sessions lasting 20–30 min each. Sessions 1 and 2 consisted of matching pictures with specific emotions. The pictures used depicted manufactured objects or animals in session 1 and actions in session 2. Responses were to be given using “smiley” cards. The verbal expression of emotions and elicitation of their causes were encouraged. Sessions 3 and 4 focused more specifically on language and on the verbalization of emotions. In session 3, children were invited to verbalize the emotion of their teacher, who expressed a sentence using different intonations. They were also invited to associate musical extracts with emotional states. Session 4 used images with characters presented in a certain context. The children and their teacher were engaged in a conversation in order to improve their comprehension of each other’s emotions. Children were asked to express both the feelings of the characters and the causes of their feelings, and the teacher wrote what the children said in order to create a written trace of child elaboration.

Sequence 3 (expression of emotions) aimed to experiment with the expression of emotions through the following different ways: the face, voice, body postures, and movements. The sequence included three distinct sessions, lasting 30–40 min each, which, respectively, focused on the following: (1) facial expression in session 1 (mimes and emotion poses, which were captured by a camera); (2) body language in session 2 (the faces being masked); and (3) verbal expression (expressions through intonations). This sequence was designed in connection with the data showing that the expression of emotions in young children can improve their communication abilities [29].

### 3.4. Measures

#### 3.4.1. Emotion Abilities

**Figure 1:**
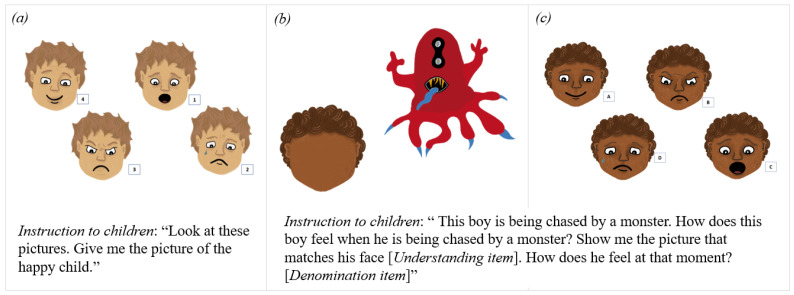
Example of items for the identification, understanding, and denomination emotion tasks. Panel (**a**): Example of an identification item. Panel (**b**): Presentation card for an example of understanding (first question in the instruction) and denomination (second question in the instruction) items. Panel (**c**): Response card for an example of understanding and denomination items.

The evaluation of emotional abilities was performed using three tasks in which children had to process the primary emotions of joy, sadness, fear, and anger (see Figure 1). The emotion-related tasks were adapted from previous work to measure young children’s emotional abilities [10]. Other emotional abilities tests exist, such as the Emotional Intelligence Test [37], but we needed a short test adapted for administration in a typical classroom setting. The first task (identification task) aimed at assessing emotional recognition. Four items—one for each emotion—were presented. In each item, children were presented with four pictures of the same face, respectively, depicting joy, sadness, fear, and anger. They were asked to point out an emotion labeled by the experimenter (for example, “show me the picture of the child who is sad”). The second and third tasks assessed the understanding of the cause of the emotion and the labeling of the emotion, respectively. In each item, children were presented with a card showing an event (for example, a boy receiving a present for his birthday), which was verbally described by the experimenter. In the second task (an understanding task), children were asked to point out the pictured face corresponding to the emotion felt by the character (“How does the boy who received a present for his birthday feel?”) among four pictures of the same face, respectively, depicting joy, sadness, fear, and anger. In the third task (the denomination task), children were asked to label the pointed-out emotion (“what does he feel at that moment?”). Four items were presented—one for each emotion. In each task, the positions of the pictures depicting joy, sadness, fear, and anger varied from one item to another. Each of the tasks was scored with 4 points (1 point for each correct response). The total emotion score was 12. It was transformed into the percentage of correct responses.

#### 3.4.2. Language Comprehension Abilities

The evaluation of language comprehension was performed using two tasks from two standardized tests in lexical and syntactic comprehension. The first task was adapted from the “Echelle de Vocabulaire en Images de Peabody, EVIP” [38] (French version of the Peabody Picture Vocabulary Test). Six items were selected for our adapted task, varying according to their complexity, to avoid floor and ceiling effects at each age level. In each item, the experimenter pronounced a word, saying: “Which image represents the word __?” Children were asked to indicate, among the four images, which one corresponded to the word pronounced by the experimenter. The second task was adapted from the “Epreuve de Compréhension Syntaxico-Sémantique, E.CO.S.SE” [39] aimed at assessing syntactic understanding. Six items were selected in the same manner as described above for lexical comprehension. Children were asked to indicate, among the four images, which one corresponded to what the experimenter said. One point was given for each correct response, so the highest language score was 12. This was then transformed into the percentage of correct responses.

#### 3.4.3. Mathematics Abilities

The assessment of mathematic abilities was performed using two experimental tasks designed through collaborative work between the teachers and researchers: a numerical task and a reasoning task (adapted to Cavadini et al. [10]). The first task focused on numerical processes with quantities from 1 to 6: subitizing, counting, addition, comparison, and mathematics vocabulary (more than, less than, as much). The subtotal score was 15. The second task assessed reasoning and, more specifically, the comprehension of algorithms or patterning (alignment of a sequence of cards respecting a detected regularity). Different cards varying in shape (four shapes) and color (four colors) were used. In each item, the experimenter started by showing the child the construction of a sequence of cards. Children had to continue the sequence from the initial construction. Four items of increasing difficulty were presented. The subtotal score was 9. The maximal score for the mathematics task was 24 points. We computed the rate of success as a percentage.

## 4. Data Analysis

Statistical analyses were computed using IMB SPSS Statistics 28.0 computer software. First, we conducted correlation analyses (partial Pearson’s *r*) between the emotional ability total score, language comprehension, and mathematics performance scores (in percentages). 

To analyze the direct global training effect on emotions, we ran a 2(Time: pretest, posttest) × 2(Group: intervention, non-intervention) × 3(Level: 1, 2, 3) ANOVA on the total emotional ability score. To better understand the direct effect of emotion training, we also conducted three 2(Time: pretest, posttest) × 2(Group: intervention, non-intervention) × 3(Level: 1, 2, 3) ANOVAs, respectively, on the score for each emotion task (identification, understanding, and denomination). We used the partial eta squared to measure the effect sizes.

Finally, to investigate the transfer effect of emotion training on language comprehension and mathematic abilities, two 2(Time: pretest, posttest) × 2(Group: intervention, non-intervention) × 3(Level: 1, 2, 3) ANOVAs were performed, respectively, on the total language score and on the total mathematics score. We used the partial ETA squared to measure the effect sizes.

## 5. Results

### 5.1. Direct Training Effect on Emotion Abilities

First, in order to have only one total score for emotional abilities, we checked the intercorrelation of the three emotion tasks. The scores obtained in the pretest on the three emotion tasks (identification, understanding, and denomination) were significantly and positively correlated. The correlations between identification and understanding (*r* = 0.46, *p* < 0.001) and between identification and denomination (*r* = 0.47, *p* < 0.001) were moderate. The correlation between understanding et denomination was strong (*r* = 0.76, *p* < 0.001). We then computed a composite score for emotional abilities with a sum of the scores from each emotion task.

Second, to analyze the direct global training effect on emotion, a 2(Time: pretest, posttest) × 2(Group: intervention, non-intervention) × 3(Level: 1, 2, 3) ANOVA was performed on the total emotional ability score. The analysis revealed a significant group effect, *F*(1519) = 9.18, *p* = 0.003, η^2^_p_ = 0.02, a significant level effect, *F*(2519) = 168.31, *p* < 0.001, η^2^_p_ = 0.39, and a significant time effect, *F*(1519) = 289.50, *p* < 0.001, η^2^_p_ = 0.34. The results also showed a significant time × level interaction, *F*(2519) = 39.61, *p* < 0.001, η^2^_p_ = 0.13, a significant time × group interaction, *F*(2519) = 7.40, *p* = 0.007, η^2^_p_ = 0.02, but no level × group interaction, *F*(2519) = 1.13, *p* = 0.33.

**Figure 2:**
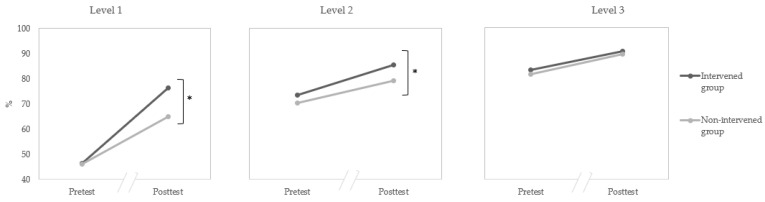
Percentage of correct responses (total score) in emotion tasks for the function of level, time, and group. The * sign means that the difference is significant (*p* < 0.05).

A significant three-way time × group × level interaction was observed, *F*(2519) = 5.05, *p* = 0.007, η^2^_p_ = 0.02 (see Figure 2). Simple effects analyses showed that, for children in Level 1, no difference between the intervention group and the non-intervention group was observed for the pretest, *F*(1169) = 0.38, *p* = 0.54, whereas children in the intervention group were significantly more efficient than the non-intervention group in the posttest, *F*(1163) = 14.56, *p* < 0.001. The results showed the same pattern for children in Level 2. No difference was observed between the intervention group and the non-intervention group in the pretest, *F*(1155) = 0.93, *p* = 0.34), whereas children in the intervention group were significantly more efficient than the non-intervention group in the posttest, *F*(1138) = 5.66, *p* = 0.02. However, no training effect was observed for children in Level 3. No difference was observed between the intervention group and the non-intervention group in the pretest, *F*(1238) = 1.25, *p* = 0.26, and in the posttest, *F*(1228) = 0.25, *p* = 0.62. When the IPS (the measure of socioeconomic status) was entered into the analysis, this interaction remained significant.

### 5.2. Direct Training Effect on Each Emotion Task

Table 2 shows the percentage of correct responses (the mean, SD) on the three emotion tasks for the function of group, level, and time. To better understand the effect of emotion training, we conducted three 2(Time: pretest, posttest) × 2(Group: intervention, non-intervention) × 3(Level: 1, 2, 3) ANOVAs, respectively, on the score for each emotion task.

### 5.3. Identification Task

An analysis revealed a significant group effect, *F*(1523) = 26.40, *p* < 0.001, η^2^_p_ = 0.05, a significant Level effect, *F*(2523) = 58.61, *p* < 0.001, η^2^_p_ = 0.18, and a significant time effect, *F*(1523) = 82.78, *p* < 0.001, η^2^_p_ = 0.14. The results also showed a significant time × level interaction, *F*(2523) = 26.86, *p* < 0.001, η^2^_p_ = 0.09, but no time × group interaction, *F*(2523) = 0.20, *p* = 0.66, no level × group interaction, *F*(2523) = 2.62, *p* = 0.07, and no time × level × group interaction, *F*(2523) = 1.64, *p* = 0.20. In this task, when assessing emotion identification, the differences between the posttest and the pretest scores did not differ between the intervention and the non-intervention groups, whatever the level. When the IPS was entered into the analysis, the effects and interactions remained unchanged.

### 5.4. Understanding Task

The analysis revealed no group effect, *F*(1523) = 0.10, *p* = 0.75, but a significant level effect, *F*(2523) = 141.20, *p* < 0.001, η^2^_p_ = 0.35, and a significant time effect, *F*(1523) = 120.22, *p* < 0.001, η^2^_p_ = 0.19. The results also showed a significant time × level interaction, *F*(2523) = 18.59, *p* < 0.001, η^2^_p_ = 0.19, a significant time × group interaction, *F*(2, 523) = 5.11, *p* = 0.02, η^2^_p_ = 0.01, but no level × group interaction, *F*(2, 523) = 0.73, *p* = 0.49. When the IPS was entered into the analysis, the time × group interaction remained significant. 

A significant three-way time × group × level interaction was observed, *F*(2, 523) = 2.95, *p* = 0.05, η^2^_p_ = 0.01. Simple effects analyses showed that, for children in Level 1, no difference between the intervention group and the non-intervention group was seen in the pretest *F*(1169) = 1.43, *p* = 0.23, whereas children in the intervention group were significantly more efficient than the non-intervention group in the posttest, *F*(1164) = 6.58, *p* = 0.01. However, in Level 2, no difference was observed between the intervention group and the non-intervention group in the pretest, *F*(1155) = 0.008, *p* = 0.93, or in the posttest, *F*(1140) = 0.16, *p* = 0.69. The results showed the same pattern for Level 3 children. No difference was observed between the intervention group and the non-intervention group in the pretest, *F*(1238) = 0.03, *p* = 0.86, or in the posttest, *F*(1229) = 0.11, *p* = 0.74. When the IPS was entered into the analysis, this interaction disappeared.

### 5.5. Denomination Task

The analysis revealed a significant group effect, *F*(1519) = 7.57, *p* = 0.006, η^2^_p_ = 0.01, a significant level effect, *F*(2519) = 144.64, *p* < 0.001, η^2^_p_ = 0.36, and a significant time effect, *F*(1519) = 265.18, *p* < 0.001, η^2^_p_ = 0.34. The results also showed a significant time × level interaction, *F*(2519) = 20.95, *p* < 0.001, η^2^_p_ = 0.08, a significant time × group interaction, *F*(2519) = 8.82, *p* = 0.003, η^2^_p_ = 0.02, but no level × group interaction, *F*(2519) = 0.83, *p* = 0.43. 

A significant three-way time × group × level interaction was observed, *F*(2519) = 4.33, *p* = 0.01, η^2^_p_ = 0.02. Simple effects analyses showed that, for children in Level 1, no difference between the intervention group and the non-intervention group was observed in the pretest, *F*(1169) = 3.23, *p* = 0.07, whereas children in the intervention group were significantly better than the non-intervention group in the posttest, *F*(1169) = 9.46, *p* = 0.002. The results showed the same pattern for children in Level 2. No difference was observed between the intervention group and the non-intervention group in the pretest, *F*(1155) = 0.95, *p* = 0.33, whereas children in the intervention group were significantly better than the non-intervention group in the posttest, *F*(1138) = 7.69, *p* = 0.006. However, no training effect was observed for children in Level 3. No difference was observed between the intervention group and the non-intervention group in the pretest *F*(1238) = 0.33, *p* = 0.56, (*p* = 0.51) or in the posttest, *F*(1228) = 0.65, *p* = 0.42. When the IPS was entered into the analysis, this interaction remained significant.

### 5.6. Relation between Emotion, Language, and Mathematics Abilities (Transfer Effects)

Table 3 shows the percentage of correct responses (the mean, SD) for the language comprehension and mathematics tasks for the function of group, time, and level.

First, the correlations between the total emotion and language scores (*r* = 0.60, *p* < 0.001) and between the total emotion and mathematics scores (*r* = 0.66, *p* < 0.001) were significant, positive, and moderate.

Second, to investigate the transfer effect of emotion training on language and mathematics abilities, two 2(Time: pretest, posttest) × 2(Group: intervention, non-intervention) × 3(Level: 1, 2, 3) ANOVAs were performed, respectively, on the total language score and on the total mathematics score.

Concerning the language abilities, the analysis revealed a significant group effect, *F*(1, 351) = 40.88, *p* < 0.001, η^2^_p_ = 0.10, a significant level effect, *F*(2, 351) = 88.77, *p* < 0.001, η^2^_p_ = 0.34, and a significant time effect, *F*(1, 351) = 118.12, *p* < 0.001, η^2^_p_ = 0.25. The results also showed a significant time × level interaction, *F*(2, 351) = 2.99, *p* = 0.05, η^2^_p_ = 0.02, but no time × group interaction, *F*(2, 351) = 1.37, *p* = 0.24, no level × group interaction, *F*(2, 351) = 0.35, *p* = 0.71, and no time × level × group interaction, *F*(2, 351) = 0.97, *p* = 0.38. The results were similar when the dependent variables were the word or the sentence comprehension abilities. In summary, the differences between the posttest and the pretest scores in language did not differ between the intervention and the non-intervention groups, whatever the level.

Concerning the mathematics ability, the analysis revealed a significant group effect, *F*(1396) = 13.51, *p* < 0.001, η^2^_p_ = 0.03, a significant level effect, *F*(2396) = 305.27, *p* < 0.001, η^2^_p_ = 0.61, and a significant time effect, *F*(1396) = 344.31, *p* < 0.001, η^2^_p_ = 0.47. The results also showed a significant time × level interaction, *F*(2396) = 5.06, *p* = 0.007, η^2^_p_ = 0.03, but no time × group interaction, *F*(2396) = 2.07, *p* = 0.15, no level × group interaction, *F*(2396) = 1.16, *p* = 0.31, and no time × level × group interaction, *F*(2396) = 0.14, *p* = 0.87. The results were similar when the dependent variables were the numerical or reasoning (patterning) abilities. In summary, the differences between the posttest and the pretest scores in mathematics did not differ between the intervention and the non-intervention groups, whatever the level.

## 6. Discussion

### 6.1. Interpretation of Results

The main objective of our study was to explore whether the training of emotional ability in preschool and kindergarten children, based on research data and adapted to usual classroom practices, could impact their emotional abilities. Supporting our hypothesis, our training positively influenced the score on emotional abilities. Globally, the fact that emotional abilities improved more in the intervention group than in the non-intervention group between the pre- and posttest showed that the current training was efficient. Subsequent analyses showed, however, that the observed improvement depended on the nature of the emotion task (all emotions being combined) and interaction with age. More specifically, the effect was demonstrated in 3-year-old children (Level 1) in tasks that evaluated emotional understanding with a nonverbal response (pointing to one of the four illustrations representing facial expressions) and emotional denomination with a verbal response (labeling the emotion). The effect was also demonstrated in 4–year-old children (Level 2) when they were asked to label the emotion (denomination). The training was not demonstrated to be efficient in the identification task, where children were asked to point to the emotional facial expression corresponding to the emotional word pronounced by the experimenter. In 5-year-old children (Level 3), the training effect did not appear in any of the tasks.

These findings are probably linked to the nature of the training carried out. In sequences 2 and 3, the children had the opportunity to analyze the contextual elements that could be the origin of an emotion and to make inferences about the nature of emotional states. They were also encouraged to express their emotions by modulating their voice, the expression on their face, and their body posture. This active engagement through various behaviors, which required verbal and non-verbal expression, helped children to develop abilities related to emotion comprehension. The lack of the training effect on the emotion identification task was surprising in view of the activities proposed during sequence 1, which focused on emotion identification. An explanation was the ceiling effect of the children in the identification task (a mean of 91.36% across the three levels; 75.33% in Level 1, 90.85% in Level 2, and 97.05% in Level 3). Even the youngest children entered preschool with basic emotional abilities, such as those involving the identification of primary emotions. A future perspective should be to assess if our emotion training is efficient for 3-year-old children who have not acquired these basic emotional identification skills or for children with emotional impairment (for example, children with autism spectrum disorder). Furthermore, we did not show any positive effect on the training on 5-year-old children (Level 3). A possible explanation is that participants already showed a high level of performance in the three tasks. This could be due to the fact that our tasks had to be adjusted to the abilities of children aged from 3 to 6, which may have led to ceiling effects in the oldest age group.

In summary, our results support the idea that it is possible to improve young children’s emotional abilities through training co-constructed by researchers and teachers, and that include relevant activities carried out in the classroom. Our result also extends the positive effects of a program on emotion abilities observed by Giménez-Dasí et al. [29] in 4–5-year-old children with special needs (see also Sprung et al. [40] for a meta-analysis).

The second objective was to assess whether children’s emotional abilities were related to their language comprehension and mathematics abilities in the pretest period. The results showed a significant, positive, and moderate correlation between emotional ability and the two academic performances. Our results are, thus, consistent with those of previous studies that show a correlation between emotional abilities and mathematics abilities [9,10] and extend this knowledge to the relationship between emotional abilities and language comprehension abilities. While previous studies showed an association between emotion and language comprehension in 3-years-olds [32] and between emotion and language expression in 3–5-year-old children [33], our study demonstrated a link between emotion and language comprehension in 3–6-year-old children.

In addition to studying the existing link between emotional abilities and academic performance, emphasis was placed in our study on the causal direction of this link (objective 3). Our results did not provide evidence of a transfer effect of emotion training on mathematics and language. Children in the intervention group indeed did not increase their performance in mathematics and language comprehension between the pretest and posttest more than children in the non-intervention group. The positive effect of the training (which was demonstrated in the youngest age groups on emotion comprehension) did not transfer to tasks involving these contents of learning. Accordingly, our study failed to demonstrate that emotional abilities are a determinant of academic performances when these performances concern the early learning abilities of mathematics and language comprehension.

In the educational literature, one of the issues generally discussed about transfer concerns the possibility of far transfer. A “far transfer” is defined as knowledge or skills acquired in a specific domain that improves related performance in a dissimilar domain [41]. Conversely, a “near transfer” occurs within the same or a close domain. However, there are many grey areas regarding the near or distant nature of certain transfers, and it may be more of a continuum. There is no consensus within the scientific community as to whether distant transfers are possible. Some works claim that they are very difficult, if not impossible, to achieve, while others consider that the question is rather one of the conditions that allow it to happen, as it is a question of being able to evoke a previously studied situation despite major differences in context and content. The lack of consensus on the possibility of remote transfer is partly due to a lack of a clear, shared definition, notably on the nature of the transferred skill and on the semantic distance between the training context and the transfer context.

If we accept the possibility of a transfer, the lack of the training transfer effect in our study could be discussed in several ways. There may be no causal link between emotional abilities in mathematics and language comprehension abilities that are built at preschool and kindergarten levels. An initial explanation could be that the transfer effect of the training could be a long-term effect and could, therefore, be seen later in development. This hypothesis needs to be tested with a similar study with a follow-up. A second explanation could concern a gap between the type of emotional abilities targeted in the current training and the type of emotional abilities that could be causally related to mathematics and language comprehension abilities. In fact, given the age of the youngest children, our training focused on basic emotional abilities, such as identifying emotions, understanding children’s own emotions and others’ emotions in situations, and naming emotions. One may explain that emotional regulation could be rather causally linked with mathematics and language comprehension. The ability to regulate emotions refers to the ability to change the nature or intensity, duration, or expression of emotions. Emotional regulation abilities can support academic learning in promoting peer acceptance, school adaptation, communication with the teacher and the other students, anxiety regulation, and academic performance. This hypothesis should be verified in a future study.

### 6.2. Strengths and Limitations

In addition to the large sample of children, the primary strength of the present study was that the training and the tasks were co-constructed through a collaboration between researchers and teachers. In this approach, researchers transmitted their scientific knowledge so that teachers could appropriate it. Teachers engaged in the co-construction of training met their pedagogical constraints and were consistent with scientific knowledge and research findings. This approach constituted a strength of our research in guaranteeing external validity. What was proposed was the development of realistic training adapted to the daily practice of the class. Also, we chose a collaborative approach since it was likely to have indirect benefits for teachers and their practices. The co-construction of the training could allow them, for example, to better understand the basis of emotional development and to develop their own emotional abilities [42]. It can make them aware of the functional role of emotion in school activities, which promotes reflection on learning methods and contents. Being aware of how to evaluate the effect of training may also allow teachers to better understand the impacts of their professional actions.

Two main limitations to this study can be noted. First, we did not show any positive effect of the training in 5-year-old children (Level 3) partly because they already showed a high level of performance in emotional identification, understanding, and denomination. Consequently, our next objective is to build training sequences and evaluation tasks based on emotional abilities adapted to 5–6-year-old children (Level 3). For this, the training and the evaluation tasks should focus on more elaborate contents, with a diversification of the nature of emotions (inclusion of secondary or complex emotions, such as shame, culpability, etc.) and sequences on emotion regulation. Secondly, we did not measure the children’s engagement during the sessions nor their adhesion to the training (concerning the teachers, we assumed a priori a strong adhesion given their voluntary investment in the research). These implementation elements should be verified in a future study.

## 7. Conclusions

The central question of this study was whether training adapted to usual classroom practices could help children improve their emotional abilities. Given the research findings on the nature and development of young children’s emotional abilities, the training focused on the abilities of the identification, understanding, and denomination of emotions. These abilities were trained within the framework of an active pedagogy, allowing children to experience knowledge through numerous activities. Through the training, language and peer interactions were encouraged. The following two main aspects were targeted in our collaborative approach: (a) the nature of the activities to be implemented as a function of research data on the development of emotional abilities; (b) the format of these activities for their integration into a usual classroom context. Our interactions with teachers resulted in the co-construction of training, which was based on the scientific literature and adapted to classroom practices, which contributed to improving the emotional abilities of 3-year-old children (Level 1) and, to a lesser extent, in 4-year-old children (Level 2). Finally, the basic emotional abilities (identification, understanding, and denomination of primary emotions) were correlated to mathematics and language comprehension abilities, but the nature of this link was not demonstrated to be causal. Emotional regulation may be rather causally connected with mathematics and language abilities, but this hypothesis should be verified in a future study.

## Figures and Tables

**Table 1:** Participants.

		Level 1 (*n* = 175)		Level 2 (*n* = 160)		Level 3 (*n* = 250)	
		Intervention Group	Non-Intervention Group	Intervention Group	Non-Intervention Group	Intervention Group	Non-Intervention Group
*n* =		104	71	70	90	142	108
Gender	Boys	48%	48%	56%	50%	49%	48%
	Girls	52%	52%	44%	50%	51%	52%
IPS: *M* (SD)		109.87 (6.23)	110.41 (12.09)	109.87 (7.38)	110.22 (10.60)	104.18 (12.36)	97.17 (11.24)

**Table 2:** Percentage of correct responses (the mean, SD) in the three emotion tasks for the function of level, time, and group.

		Level 1 (*n* = 175)		Level 2 (*n* = 160)		Level 3 (*n* = 250)		Total (*n* = 585)	
		Intervention Group	Non-Intervention Group	Intervention Group	Non-Intervention Group	Intervention Group	Non-Intervention Group	Intervention Group	Non-Intervention Group
Identification	Pretest	79.12 (25.94)	71.54 (30.26)	93.18 (15.53)	88.51 (19.49)	99.26 (4.25)	94.84 (11.11)	91.36 (18.88)	86,26 (23.96)
	Posttest	96.65 (11.20)	86.54 (23.42)	100.00 (0.00)	92.23 (15.99)	99.44 (3.70)	98.37 (8.92)	98.66 (6.98)	93.07 (16.99)
Understanding	Pretest	40.46 (25.37)	43.85 (29.33)	70.45 (27.70)	72.64 (25.57)	81.11 (17.40)	82.07 (19.02)	65.52 (28.87)	68.29 (28.99)
	Posttest	70.36 (25.60)	60.38 (23.76)	80.30 (21.27)	80.07 (24.12)	88.52 (16.38)	89.13 (17.11)	80.79 (22.24)	78.14 (24.41)
Denomination	Pretest	18.49 (23.85)	23.08 (25.89)	56.25 (34.21)	50.34 (30.83)	69.78 (22.96)	67.39 (26.67)	50.09 (34.47)	49.46 (33.09)
	Posttest	61.72 (30.12)	47.69 (29.22)	76.95 (22.40)	65.88 (28.24)	83.96 (17.71)	81.25 (23.62)	75.17 (25.30)	66.88 (29.30)

**Table 3:** Percentage of correct responses (the mean, SD) for the language and mathematics tasks for the function of level, time, and group.

		Level 1 (*n* = 114)		Level 2 (*n* = 100)		Level 3 (*n* = 188)		Total (*n* = 402)	
		Intervention Group	Non-Intervention Group	Intervention Group	Non-Intervention Group	Intervention Group	Non-Intervention Group	Intervention Group	Non-Intervention Group
Language	Pretest	52.52 (20.16)	41.22 (18.21)	71.43 (15.02)	58.97 (23.37)	80.42 (14.92)	73.43 (20.68)	66.74 (21.38)	59.82 (24.62)
	Posttest	66.06 (16.94)	55.41 (20.71)	81.46 (13.85)	67.09 (21.46)	91.97 (8.58)	79.40 (23.73)	78.80 (17.91)	68.80 (24.21)
Mathematics	Pretest	17.56 (11.81)	15.48 (9.78)	50.07 (24.94)	40.09 (22.42)	75.22 (17.72)	70.37 (25.06)	52.97 (30.58)	46.23 (31.06)
	Posttest	36.34 (19.31)	30.48 (19.06)	70.41 (22.88)	58.22 (27.98)	88.87 (9.47)	82.41 (18.70)	69.62 (27.68)	60.88 (30.56)

## Data Availability

Publicly available datasets were analyzed in this study. This data can be found here: https://osf.io/w3xmj/, (accessed on 16 November 2023).
